# The Utility of (1→3)-β-D-Glucan Testing in the Diagnosis of Coccidioidomycosis in Hospitalized Immunocompromised Patients

**DOI:** 10.3390/jof8080768

**Published:** 2022-07-25

**Authors:** Mohanad M. Al-Obaidi, Parham Ayazi, Aishan Shi, Matthew Campanella, Elizabeth Connick, Tirdad T. Zangeneh

**Affiliations:** Division of Infectious Diseases, University of Arizona College of Medicine-Tucson, Tucson, AZ 85724, USA; parhamayazi@yahoo.com (P.A.); ashi1@arizona.edu (A.S.); mcampanella@email.arizona.edu (M.C.); connicke@arizona.edu (E.C.); tzangeneh@arizona.edu (T.T.Z.)

**Keywords:** coccidioidomycosis, (1→3)-β-D-glucan, fungal diagnostics, immunocompromised

## Abstract

Coccidioidomycosis is a fungal infection endemic to the Southwestern United States which is associated with high morbidity and mortality in immunocompromised hosts. Serology is the main diagnostic tool, although less sensitive among immunocompromised hosts. (1→3)-β-D-glucan (BDG) is a non-specific fungal diagnostic test that may identify suspected coccidioidomycosis and other invasive fungal infections. We retrospectively investigated the utility of BDG between 2017 and 2021 in immunocompromised hosts with positive *Coccidioides* spp. cultures at our institutions. During the study period, there were 368 patients with positive cultures for *Coccidioides* spp.; among those, 28 patients were immunocompromised hosts, had both *Coccidioides* serology and BDG results available, and met other inclusion and exclusion criteria. Half of the patients had positive *Coccidioides* serology, and 57% had a positive BDG ≥ 80 pg/mL. Twenty-three (82%) had at least one positive test during their hospitalization. Among immunocompromised hosts with suspicion for coccidioidomycosis, the combination of *Coccidioides* serology and BDG can be useful in the initial work up and the timely administration of appropriate antifungal therapy. However, both tests failed to diagnose many cases, underscoring the need for better diagnostic techniques for identifying coccidioidomycosis in this population.

## 1. Introduction

Coccidioidomycosis is a fungal infection endemic to the Southwestern United States, Mexico, and parts of Central and South America. It is caused by two genetically distinct species of soil-inhabiting molds, *Coccidioides immitis* and *Coccidioides posadasii* [1]. Coccidioidomycosis is associated with increased morbidity and mortality in immunocompromised hosts [2,3,4]. Because fungal cultures and/or pathology are not rapidly available and may cause a delay in the diagnosis, *Coccidioides* serologic tests are the mainstay of diagnosis. However, the sensitivity of serologic tests is lower in immunocompromised hosts than in immunocompetent individuals [5,6].

Besides serologies, diagnosis of coccidioidomycosis can be made by detecting *Coccidioides* galactomannan antigen or the fungal polysaccharide (1→3)-β-D-glucan (BDG); however, BDG has low sensitivity in immunocompetent patients with coccidioidomycosis [7]. In a study where 12 serum samples were positive by *Coccidioides* galactomannan antigen tests, 92% had a positive serum BDG [8]. Moreover, the combination of different serologic tests was shown to increase the sensitivity of coccidioidomycosis diagnosis in the immunocompromised hosts [5]. The utility of BDG alone or in combination with serology for diagnosing coccidioidomycosis among immunocompromised hosts is unknown. We evaluated the sensitivity of BDG alone and in combination with serology for diagnosing coccidioidomycosis among hospitalized immunocompromised hosts.

## 2. Materials and Methods

A retrospective study of patients hospitalized between 1 October 2017 to 30 September 2021 at three of our hospitals in Arizona was performed. Inclusion criteria were patients ≥ 18 years of age with positive *Coccidioides* spp. cultures, who had *Coccidioides* serology and serum BDG testing within two weeks of the culture collection. Immunocompromised hosts included patients with malignancies on chemotherapy, solid organ transplant (SOT), hematopoietic stem cell transplant (HSCT), and those receiving high-dose steroids (pulse dose steroid, 20 mg daily for ≥14 days, or dexamethasone for 10 days or more) and/or other immunosuppressive agents. Patients with other invasive fungal infections (IFI), such as *Pneumocystis jirovecii* pneumonia (PJP), aspergillosis, and invasive candidiasis, were excluded. Patients were also excluded if they received intravenous immunoglobulin (IVIG), cytomegalovirus immunoglobulin, albumin, or fresh frozen plasma within 30 days from BDG testing to avoid false-positive BDG [9]. Additional data, including demographics, immunosuppressive conditions, and medications, were collected by medical record review.

*Coccidioides* spp. growth on Sabouraud dextrose fungal media was utilized and confirmed using AccuProbe^®^ Hologic *Coccidioides* DNA probe. Serum BDG was performed using Cape Cod, Inc Fungitell™ (reference range ≤ 31 pg/mL and ≥500 pg/mL), with ≥80 pg/mL considered as positive, according to the manufacturer’s instructions. Serologic testing consisted of enzyme immunoassay (EIA) using IMMY OMEGA Cocci Ab EIA Test Kit, immunodiffusion (IMDF) using IMMY, and complement fixation (CF) using Meridian Bioscience with IMMY CF-Fungal Antigens and controls. Similar to previous studies, indeterminate *Coccidioides* serology results were considered negative [5,10].

Chi-square tests were used to compare categorical variables. For variables with nonparametric distributions, Wilcoxon rank-sum and Kruskal–Wallis tests were used, when indicated. Two-sided tests were used with a *p*-value < 0.05 considered statistically significant.

## 3. Results

A total of 368 patients with positive *Coccidioides* spp. cultures were identified, and 78 (21%) met the study’s criteria of immunocompromised hosts. Fifty immunocompromised hosts (64%) were excluded for the following reasons: 23 received IVIG, albumin, or fresh frozen plasma, 23 did not have *Coccidioides* serology and/or BDG results available, two had a BDG order >14 days from fungal culture collection and two had PJP. None of the included patients had a history of recent abdominal or bowel surgeries. Twenty-eight immunocompromised hosts with coccidioidomycosis met inclusion criteria for the final analysis (Figure S1). Clinical characteristics are shown in Table 1. The median age was 58 years, and the majority were white males. Twenty-four (86%) positive cultures were isolated from a pulmonary source, two from blood, one from an extremity abscess, and one from the cerebrospinal fluid. The majority of patients had SOT or malignancies.

Sixteen (57%) of the immunocompromised hosts had a positive BDG (Table 2). Of patients with detectable BDG levels below the threshold for a positive result, two had positive serology, and three had negative serology (Table 2). The median time from serum BDG sample collection to fungus culture sample collection was 3.9 days (IQR 2.2–7.5). Among positive BDG, the median BDG level was 301 pg/mL (IQR, 130–500 pg/mL). Fourteen patients (50%) had positive *Coccidioides* serology (Table S1), with 12 (86%) positive by IgG EIA, five (36%) positive by EIA IgM, 12 (86%) positive by IMDF IgG, and 10 (71%) positive by IMDF IgM. Thirteen patients had an available CF titer of which two had a titer of 1:256, one each with 1:64, 1:16, and 1:8, two with a titer of 1:4, and six had a titer < 1:2. There were no statistically significant differences between positive and negative BDG or *Coccidioides* serology in terms of demographics, type of immunosuppression, or culture source (Table S2). Among SOT (*n* = 12) patients, however, positive *Coccidioides* serology was more frequent among liver transplant recipients (four of five had positive tests) than other organ transplant recipients in whom none of seven had a positive serologic result.

Twenty-three (82%) patients had a positive test for either *Coccidioides* serology and/or serum BDG, including eight (29%) who had a positive test for both. Six (21%) had positive *Coccidioides* serology with negative BDG and eight (29%) had positive BDG with negative serology. There were no statistically significant differences in terms of demographics, type of immunosuppression, or culture source between those with positive or negative results with combined testing

## 4. Discussion

Diagnosis of coccidioidomycosis in immunocompromised hosts is challenging as serologic tests are frequently negative [6]. BDG has previously been shown to be positive in many IFI, including immunocompetent adults diagnosed with coccidioidomycosis [7]. This is the first study to explore the utility of BDG alone and in combination with *Coccidioides* serology in the diagnosis of coccidioidomycosis in hospitalized immunocompromised hosts. The yield of either test alone was approximately 50%, but, in combination, 82% of the patients with coccidioidomycosis had a positive result on at least one of the tests. These findings suggest that testing by both *Coccidioides* serology and BDG in immunocompromised hosts can help in the initial diagnostic work up and may be useful when a diagnosis of coccidioidomycosis is highly suspected.

BDG’s sensitivity of 57% for coccidioidomycosis observed in the present study is not substantially different from the sensitivity of 44% previously reported in immunocompetent hosts [7]. Interestingly, BDG levels were detectable, but below the threshold for a positive result in five (18%) of the immunocompromised hosts with coccidioidomycosis (one culture from cerebrospinal fluid and four with pulmonary source), and in two of those cases *Coccidioides* serology was negative. It is recommended by the manufacturer to use the cut-off of 80 pg/mL for BDG test positivity and utility of different cut-offs levels will require further study. Prior use of immunoglobulins, albumin, and FFP has been linked to false-positive BDG results [11]. Because of this, more than half of the immunocompromised hosts with culture-proven coccidioidomycosis originally identified in this study were excluded because they had received these products. This is a significant limitation of the use of BDG in this population.

The sensitivity of serologic testing of 50% observed in the present study falls within the rates of 21% and 56% previously reported among SOT with a single serologic test [5] and is lower than the sensitivity reported in immunocompetent patients historically ranging from 70 to 95% [6,10]. In a prior study of immunocompromised hosts, the sensitivity of *Coccidioides* serology varied among different immunosuppressive conditions [6]. We observed that serologic testing was more frequently positive in liver transplant recipients compared to other SOT, which could be secondary to differences in the immunosuppression used in this group compared to other SOT patients. Importantly, the sensitivity of different commercial serologic tests varies, and newer tests, such as the MVista *Coccidioides* serologic test, have been shown to be more sensitive than older serologic tests [10]. Further study of the utility of BDG in combination with newer serologic tests is warranted.

Strengths of this study include that all cases were proven coccidioidomycosis by culture. Each case was verified individually through chart review to avoid false positive BDG tests and non-coccidioidomycosis cases. This study provides real-world data on the diagnosis of coccidioidomycosis in a diverse immunocompromised population. Nevertheless, the study has several limitations. This was a retrospective study and cases were selected based upon the availability of positive cultures for *Coccidioides* spp., serology, and BDG assays, which may have introduced selection biases. Also, the number of cases was relatively small, particularly the number of cases of disseminated coccidioidomycosis, thereby limiting conclusions, specifically the relation of disseminated infection to positive BDG. While we excluded cases with possible false-positive BDG, positive BDG in the immunosuppressed patients may reflect an undiagnosed fungal infection or could be related to gastrointestinal translocation. The other limitation of the study is in eliminating cases that are diagnosed by serology or pathology; therefore, limiting the conclusion of the usefulness of BDG among non-culture positive cases. The study has also limited BDG evaluation by requiring both tests (serology and BDG) be available for the cases to be included, which reduced the sample size. Finally, based upon the study design, the specificity of BDG and serologic testing could not be determined.

In conclusion, BDG may be helpful in identifying coccidioidomycosis in hospitalized immunocompromised hosts when combined with *Coccidioides* serology. However, a definite diagnosis of coccidioidomycosis among immunocompromised hosts is warranted, and therefore the specificity of BDG should be determined. Large future studies are needed to determine the sensitivity and specificity of BDG alone and in combination with newer, more sensitive *Coccidioides* serologic testing. Lastly, both serology and BDG were negative in many immunocompromised hosts with coccidioidomycosis, underscoring the urgent need for improved diagnostic testing for this disease.

## Figures and Tables

**Table 1:** Demographic and clinical characteristics of immunocompromised patients with culture-positive coccidioidomycosis.

	Total (N = 28)
Age, median years (IQR)	58 (32–70)
Female, N (%)	12 (43)
Race/Ethnicity, N (%)	
White	15 (54)
Hispanic	9 (32)
Others	4 (14)
Immunocompromising Condition, N (%)	
SOT	12 (40)
HSCT	2 (7)
Malignancy	7 (30)
Other	7 (23)
Culture Site, N (%)	
Pulmonary	24 (86)
Extrapulmonary	4 (14)

BDG, (1→3)-β-D-glucan; HSCT, Hematopoietic Stem Cell Transplant; SOT, Solid Organ Transplant (5 liver, 3 heart, 3 kidney, and 1 combined kidney/pancreas transplant); Malignancy (3 leukemia, 2 lymphoma, 1 multiple myeloma, and one metastatic squamous cell carcinoma); Other included 5 patients with a rheumatological disease on anti-CD20 antibody, high dose steroids or biologic response modifiers; one with pulmonary fibrosis who received high dose steroids, and one with Coronavirus disease 2019 who received high dose steroids.

**Table 2:** BDG range compared to positive *Coccidioides* serology among culture positive coccidioidomycosis cases.

BDG Range, N	Positive *Coccidioides* Serology, N			
	EIA IgG	EIA IgM	IMDF IgG	IMDF IgM
≤31 (7)	3	1	4	3
32–59 (3)	2	0	2	2
60–79 (2)	0	0	0	0
≥80 (16)	7	4	6	5

BDG, (1→3)-β-D-glucan; EIA, enzyme immunoassay; IMDF, immunodiffusion. BDG level in pg/mL.

## Data Availability

Not applicable.

## Supplementary Materials

The following supporting information can be downloaded at: https://www.mdpi.com/article/10.3390/jof8080768/s1, Table S1: *Coccidioides* serology and (1→3)-β-D-glucan sensitivities, Table S2: Clinical Characteristics by tests, Figure S1: Selection of immunocompromised hosts cohort with coccidioidomycosis.
