# Supplementary material for: The Utility of (1→3)-β-D-Glucan Testing in the Diagnosis of Coccidioidomycosis in Hospitalized Immunocompromised Patients
Source: J Fungi (Basel). 2022 Jul 25;8(8):768. doi: 10.3390/jof8080768 (PMC9332557; doi:10.3390/jof8080768)
Supplement: Supplementary file 1 [file jof-08-00768-s001.zip › jof-1823602-supplementary.pdf]

Supplements:

Table S1. *Coccidioides* serology and (1→3)-β-D-glucan sensitivities.

	Positive Tests	Negative Tests
BDG, N (%)	16 / 28 (57) *	12 / 28 (43)
Coccidioides serology (%)	14 / 28 (50) *	14 / 28 (50)

*Sensitivity = (True Positives/True Positives + False Negatives).

Serology, any *Coccidioides* Enzyme Immunoassay, Immunodiffusion, or Complement Fixation; BDG, (1→3)-β-D-glucan with cut-off value of 80 pg/ml as positive test.

Table S2. Clinical Characteristics by tests.

	Positive BDG (N=16)	Negative BDG (N=12)	P-value	Positive *Coccidioides* Serology (N=14)	Negative *Coccidioides* Serology (N=14)	P-value
Age, Median (IQR)	63 (37-70)	43 (28-65)	0.26	61 (34-71)	44 (31-69)	0.37
Female, N (%)	8 (50)	4 (33)	0.38	7 (50)	5 (36)	0.45
Race/Ethnicity, N (%)			0.31			0.45
White	7 (44)	8 (67)		9 (64)	6 (43)	
Hispanic	7 (44)	2 (17)		3 (21)	6 (43)	
Others	2 (12)	2 (17)		2 (14)	2 (14)	
Immunocompromising Condition, N (%)			0.15			0.43
SOT	6 (37.5)	6 (50)		4 (29)	8 (57)	
HSCT	-	2 (17)		1 (7)	1 (7)	
Malignancy	4 (25)	3 (25)		4 (29)	3 (21)	
Other	6 (37.5)	1 (8)		5 (36)	2 (14)	
Culture Site, N (%)			0.75			1
Pulmonary	14 (88)	10 (83)		12 (86)	12 (86)	
Extrapulmonary	2 (12)[1]	2 (17)[2]		2 (14)[3]	2 (14)[4]	

[1] All from blood, [2] One cerebrospinal fluid and one musculoskeletal, [3] one blood and one musculoskeletal, [4] one blood and one cerebrospinal fluid.

BDG, (1→3)-β-D-glucan; HSCT, Hematological Stem Cell Transplant; SOT, Solid Organ Transplant (5 liver, 3 heart, 3 kidney, and 1 combined kidney/pancreas transplant); Malignancy (3 leukemia, 2 lymphoma, 1 multiple myeloma, and one metastatic squamous cell carcinoma); Other included 5 patients with a rheumatological disease on anti-CD20 antibody, high dose steroids or biologic response modifiers; one with pulmonary fibrosis who received high dose steroids, and one with Coronavirus disease 2019 who received high dose steroids.

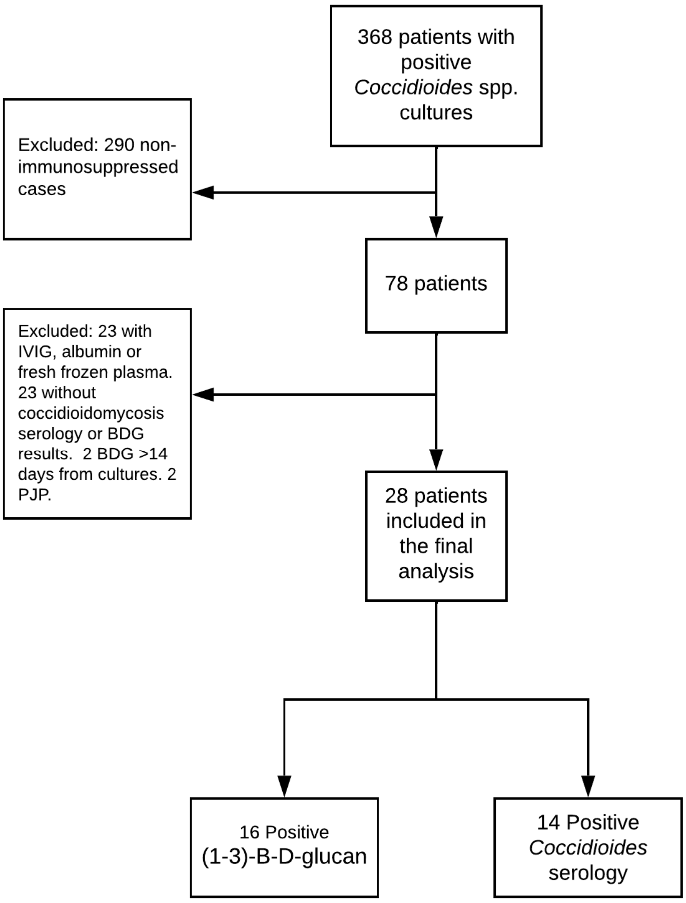

Figure S1. Selection of immunocompromised hosts cohort with coccidioidomycosis.
